# European inequalities and similarities in officially recognized dental specialties

**DOI:** 10.1186/s12903-023-02987-z

**Published:** 2023-05-11

**Authors:** Ignacio García-Espona, Eugenia García-Espona, José Antonio Alarcón, Javier Fernández-Serrano

**Affiliations:** 1 Department of Stomatology, Section of Orthodontics, School of Dentistry, University of Granada, Campus Universitario de Cartuja, s/n, Granada, 18071 Spain; 2 President of the Spanish Association of Orthodontists (AESOR), Madrid, Spain

**Keywords:** Dental specialties, Orthodontics, Oral surgery, Europe

## Abstract

**Background:**

Although the European Economic Space usually acts in a united and organized way, several main differences are found regarding the type and number of dental specialties all around this group of member states. The aim of the study is to analyse the inequalities and similarities existing between 21 European countries, highlighting the number and types of recognized dental specialties.

**Methods:**

Available official documents and webpages from 20 out of the 30 countries of which the European Economic Space is comprised plus the United Kingdom (UK), were analysed to obtain reliable data referred to dental specialties. Differences were tested with the Lorentz curve and Gini test. Additionally, a Cluster analysis was performed to obtain groups of countries with a similar pattern in the number and type of dental specialties.

**Results:**

Up to a total of 15 different specialties are officially recognized in all the analysed countries. Orthodontics (90%) and Oral Surgery (81%) are the two most frequently recognized specialties. The total global degree of inequality of the analysed countries was 40.2%. Cluster analysis differentiated three different main groups of countries according to the number and type of dental specialties.

**Conclusions:**

The situation of dental specialties in the area of the EES plus the UK exhibits an unequal organization. Cluster analysis showed 3 main clusters of countries with a similar pattern of dental specialties.

## Background

The first dental specialty, Orthodontics, was recognized in 1900 in the United States and it has been 44 years since dental specialties were established in Europe. Within the European Economic Space (EES) 25 out of the 27 countries, of which it is composed, have officially recognized dental specialties. Directives 2005/36/CE and 2013/55/EU developed the contents of three previous directives from 1978 (78/686/CEE, 78/687/CEE and 78/688/CEE) on the regulation of dental specialties. These directives were the legal support for UE professional mutual recognition and freedom of movement around the member states. Directive 2005/36/CE explicitly recognizes in annex 5.3.3. the first two professional dental specializations: Orthodontics and Oral Surgery. In order to take due account of changes in national legislation, and with a view to updating this directive, directive 2013/55/EU adds to article 35 that the Commission shall be empowered to include new dental specialties common to at least two-fifths of the Member States.

Throughout the world, regulatory bodies, set up by their country’s governments, provide specialty lists of those clinicians who have undergone a recognized postgraduate training leading to a higher specialist qualification [1].

Nowadays, the dental specialties situation remains in constant change all around Europe. Almost every country of which the EES is comprised has its own organised health system that provides conditions for the specialties regulation. While most of the member states can rely on a law regulating the dental specialties system, two countries are still omitted from these criteria: Spain and Austria, being the only ones who do not yet have this official recognition. In the case of Austria, this country is currently developing the governmental law needed to start this procedure, once they have got the parliamentary approval. Spain is currently the last country in this situation.

Furthermore, the unification of names, types and number of dental specialties in the rest of these countries is non-existent, leading to a confusing situation to identify which branches of Dentistry are recognized in which country, hindering the opportunity to move to another one and keep the same job specialization [2, 3].

Apart from this, it seems complicated to have a professional regulated system that permits us to orientate the number of dental specialists and balance each work situation in every country, because it looks inappropriate to have a lack of or a surplus of professionals in each sector of Dentistry. Moreover, these specialties help to provide a focused treatment for suitable patients, improving both the training of dentists and the quality in clinical care received by the population, building better relationships, and thus protecting the public and the specialties themselves from unqualified clinicians.

This and other observations, lead us to appreciate objective differences concerning their structure, showing health, social, economic and opportunity inequalities due to the quality of the service provided in each country, which could be partly influenced by the official recognition of dental specialties and the existence of official postgraduate studies [4–6].

Relevant information to make this comparison can be found in official documents in the national societies or associations of dentists of most of the countries. However, comparisons, even in an easy and visual way, i.e., by tables and figures, reaching the vast majority of the EES has not been reported before. Previous revised studies about this topic have analysed no more than one third of the European countries [2].

Both, from a clinical and academic perspective, including students’ perception of dental specialties [7], we considered it of great interest to evidence the objective differences that make up each unique country, looking at the number and types of dental specialties. Therefore, the aim of the present study was to analyse the inequalities and similarities existing between 20 out of the 30 countries of which the EES is comprised plus the UK, highlighting the number and types of officially recognized dental specialties.

## Methods

Available official documents and webpages, mainly linked to regulatory bodies, official college and councils and dental institutions from 20 out of the 30 countries of which the EES plus the UK, is comprised, were collected and analysed to obtain reliable data referred to dental specialties. Additional documents like annual reports were considered to extend and corroborate the public information provided (Table 1) [3, 8–31].

**Table 1:** Source of information, name of document or website address and year for every country

Country	Source of information	WEBPAGES	Documents	
			Name	Year
Austria	Österreichische Zahnärzte Kammer	https://www.zahnaerztekammer.at/zahnaerztinnen/infocenter/daten-und-zahlen	Standesm Februar	2022
Belgium	Service Publique Fédéral	https://organesdeconcertation.sante.belgique.be/fr/documents/hwf-statan-2018	Statistiques Annuelles	2019
Cyprus	Pankýpriou Odontiatrikoú Syllógou	https://www.dental.org.cy/dentists?firstname=&lastname=&city=&spid=	Database	2022
Czech Republic	Česká Stomatologická Komora	https://www.dent.cz/o-nas/rocenky/	Ročenka ČSK	2020
Denmark	Tandlaege Foreningen	https://www.tandlaegeforeningen.dk/om-tandlaegeforeningen/tandlaeger-i-tal-2021/	Tandlaeger-i-tal, Rsrapport	2021
France	Ordre National des Chirugiens-Dentistes	https://www.ordre-chirurgiens-dentistes.fr/cartographie/	Cartographie et donnés publiques	2022
Germany	Bundeszahnärztekkamer	https://www.bzaek.de/ueber-uns/daten-und-zahlen/mitgliederstatistik.html	Daten Fakten	2021
Greece	Ellinikí Odontiatrikí Omospondía	https://www.eoo.gr/eidikotites/	Database	2022
Ireland	Dental Council of Ireland	http://www.dentalcouncil.ie/dentalspecialists.php	Annual Report	2019
Italy	Ministerio della Salute	https://www.salute.gov.it/portale/temi/documenti/saluteDenti/VERBALE_GTO_8_no-vembre_2018_con_allegato.pdf	VERBALE_GTO_8_novembre_2018_con_allegato	2018
Lithuania	Lietuvos Respublikos Odontologu Rümai Lietuvos Respublikos Odontologu Rümai	https://odontologurumai.lt/lt/%C4%AFstaigoms/informacija-istaigoms https://odontologurumai.lt/lt/%C4%AFstaigoms/informacija-istaigoms	Metų veiklos ataskaita LROR Database 2012–2016	2021 2017
Luxembourg	Collège Médical	http://www.collegemedical.lu/Fr/Professionnels/	Rapport d’activité pour l’année 2020	2021
Netherlands	Koninklijke Nederlandse Maatschappij der Tandheelkunde Koninklijke Nederlandse Maatschappij der Tandheelkunde	https://www.knmt.nl/loopbaan/tandartsspecialisten https://www.knmt.nl/loopbaan/tandartsspecialisten	Verslag van het jaar 2019 Regeling specialismen tandheelkunde	2020 2015
Norway	Den Norske Tannlegeforening	https://www.tannlegeforeningen.no/om-ntf/arsrapporter-ntf.html	Ǻrsrapport 2021	2022
Poland	Polskie Towarzystwo Stomatologiczne	https://pts.net.pl/sekcje/	Database	2022
Portugal	Ordem dos Medicos Dentistas	https://www.omd.pt/especialidades/	Os números da Ordem	2021
Romania	Colegiul Medicilor Stomatologi din Románia	https://cmdr.ro/despre-cmdr/	Acte ale colegiului medicilor Dentişti din România	2012
Spain	Consejo General de Dentistas	https://www.consejodentistas.es/comunicacion/actualidad-consejo/profesion-en-cifras.html	La Profesión en Cifras 2019	2020
Sweden	Socialstyrelsen	https://sdb.socialstyrelsen.se/if_per/val.aspx	Manual of Dental Practice	2015
Switzerland	Schweizerische Zahnärzte-Gesellschaft	https://www.sso.ch/zahnarztsuche	Database	2022
United Kingdom	General Dental Council	https://www.gdc-uk.org/about-us/our-organisation/our-corporate-strategy-and-business-plans/our-annual-reports	Registration Statistical Report 2020	2021

Differences in the distribution of officially recognized dental specialties in Europe were tested with the Lorentz curve and Gini test. Additionally, a Cluster analysis was performed to obtain groups of countries with a similar pattern in the number and type of dental specialties [32].

To quantify the degree of global inequality related to the number of dental specialties of the EES analysed countries the Lorentz curve and Gini index were applied. These are two indicators commonly used to measure the unequal distribution of wealth in a society. In our context, the concept “wealth” is associated to dental wealth (number of specialties in a country), the EES acts as the society under study and each country represents an element of that society. When the relationship between the cumulative percentage of countries that make up the EES and the cumulative percentage of specialties is displayed graphically, the Lorentz curve is obtained. This curve has a very useful interpretation in order to assess inequality between countries: The closer it is to the diagonal, the more homogeneous is the number of specialties in the countries; on the contrary, if it approaches the horizontal axis, more inequality exists in the number of specialties in the different countries. One way to quantify this property is simply to measure the area between both curves and scale it, so that the result remains between 0 and 1. The result of doing this operation is known as the Gini index.

The disadvantage of the Gini index is that it only captures a part of the reality, being a simplification of it. And as such, the simplification does not explore in depth the details of which countries have greater or lesser similarity depending on the number of common specialties they share. For this reason, in order to make an assessment of the inequality between EES countries and to establish groups of inequality and similarity, we extended the study of the dental reality with a Cluster analysis^32^, which is specialized in exploring this aspect. Currently we need to define the “distance between two countries” in a way that is appropriate to the available data: Each country is represented as a binary vector with 1’s where there is a certain specialty and 0’s where it does not exist. A binary distance between two countries is then defined as the proportion of 1’s and 0’s shared by the binary vectors that represent it. Based on this definition, the distances between each pair of EES countries can be calculated through Cluster analysis so that groups and associations appear according to their similarities. All the resulting information is represented by a specialized graph called a dendrogram, which is very useful for detecting associations.

## Results

**Fig. 1:**
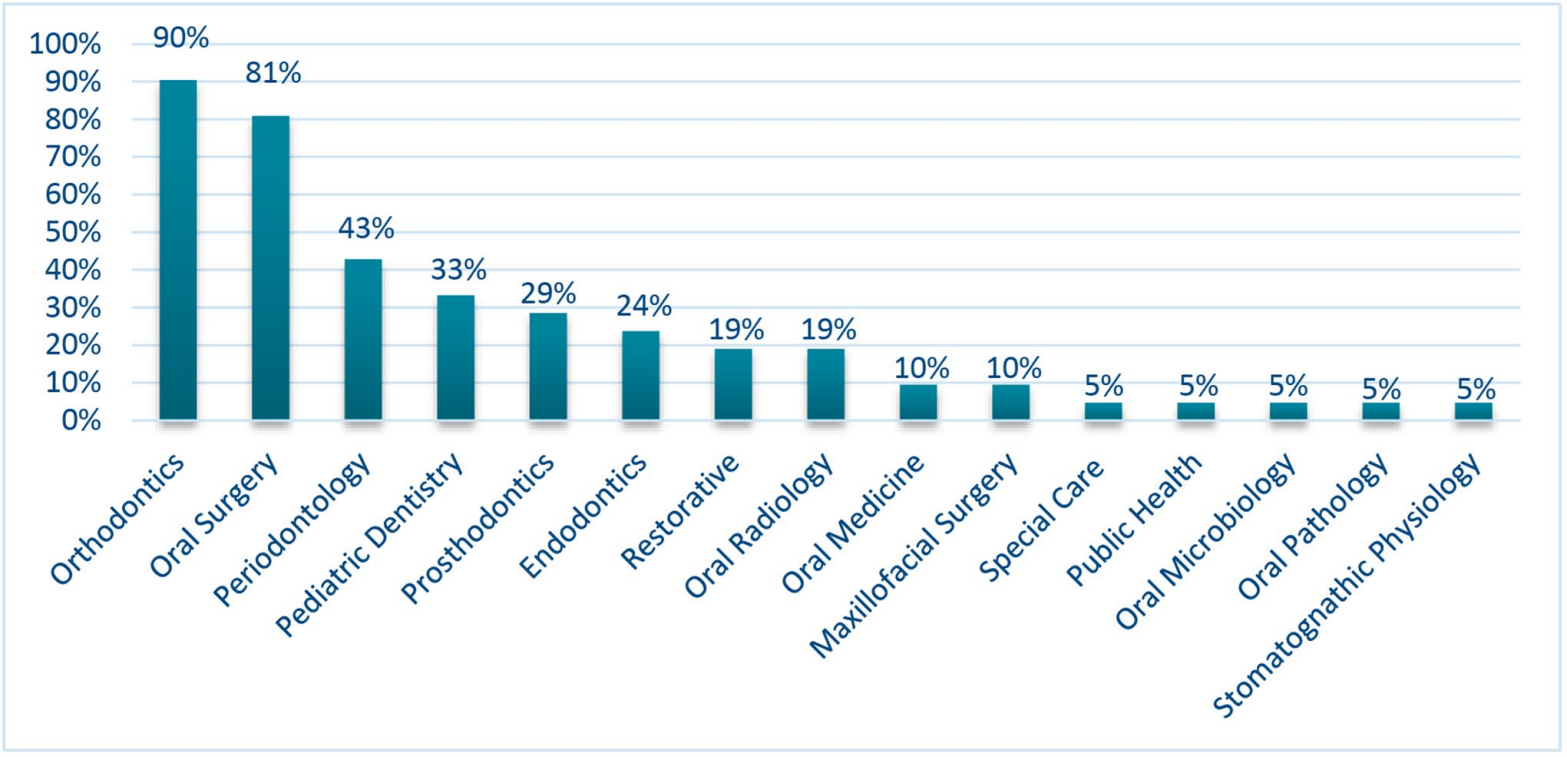
Type and percentage of officially recognized dental specialties in EES countries plus the UK

Figure 1 and Table 2 show the type, number and percentage of officially recognized dental specialties in every analysed country. Orthodontics and Oral Surgery are the most frequently recognized dental specialties of the analysed countries (90% and 81% respectively), followed by Periodontics (43%), Paediatric Dentistry (33%), Prosthodontics (29%), and Endodontics (24%).

**Table 2:** Recognized dental specialties in every analysed country

	Aus	Bel	Cyp	Cze	Den	Fra	Ger*	Gre	Ire	Ita	Lit**	Lux	Net	Nor	Pol	Por	Rom	Spa	Swe	Swi	UK	TOTAL
Orth		●	●	●	●	●	●	●	●	●	●	●	●	●	●	●	●		●	●	●	19
OSur			●	●	●	●	●	●	●	●	●	●		●	●	●	●		●	●	●	17
Per		●									●			●	●	●	●		●	●	●	9
PedD										●	●			●	●	●			●		●	7
Prost														●	●		●		●	●	●	6
End											●			●			●		●		●	5
Rest				●											●		●				●	4
Rad														●	●				●		●	4
MSur											●		●									2
OMed						●															●	2
Sp C																					●	1
PHea																					●	1
OPat																					●	1
Micr																					●	1
Phy																			●			1
	0	2	2	3	2	3	2	2	2	3	6	2	2	7	7	4	6	0	8	4	13	

Last column shows total number of countries recognizing a dental specialty.

Last row shows total number of recognized dental specialties in each country.

*Germany counts on two officially recognized dental specialties, while in some regions of the country this number reaches as many as six.

**Lithuania considers the “Orth” specialty as being two different ones: Orthodontics and Orthopedics, separately.

(Aus) Austria; (Bel) Belgium; (Cyp) Cyprus; (Cze) Czech Republic; (Den) Denmark; (Fra) France; (Ger) Germany; (Gre) Greece; (Ire) Ireland; (Ita) Italy; (Lit) Lithuania; (Lux) Luxembourg; (Net) Netherlands; (Nor) Norway; (Pol) Poland; (Por) Portugal; (Rom) Romania; (Spa) Spain; (Swe) Sweden; (Swi) Switzerland; (UK) United Kingdom

(Orth) Orthodontics and maxillofacial orthopedics; (OSur) Oral Surgery; (Per) Periodontology; (PedD) Paediatric Dentistry; (Prost) Prosthodontics; (End) Endodontics; (Rest) Restorative Dentistry/General Dentistry; (Rad) Oral Radiology; (MSur) Maxillofacial Surgery; (OMed) Oral Medicine; (Sp C) Special Care Dentistry; (PHea) Public Health; (OPat) Oral Pathology; (Micr) Oral Microbiology; (Phy) Stomatognathic Physiology

The number of recognized dental specialties was quite different as well. The most specialized country regarding the number of specialties is the UK (13 dental specialties), followed by Sweden (8), Lithuania, Norway, Poland (7), and Romania (6). Mostly of the analyzed countries recognized only 2 dental specialties. On the other hand, Austria and Spain have a total lack of officially recognized dental specialties.

The 2 most frequently recognized specialties in these countries are Orthodontics and Oral Surgery, with the exception of Belgium and Netherlands.

**Fig. 2:**
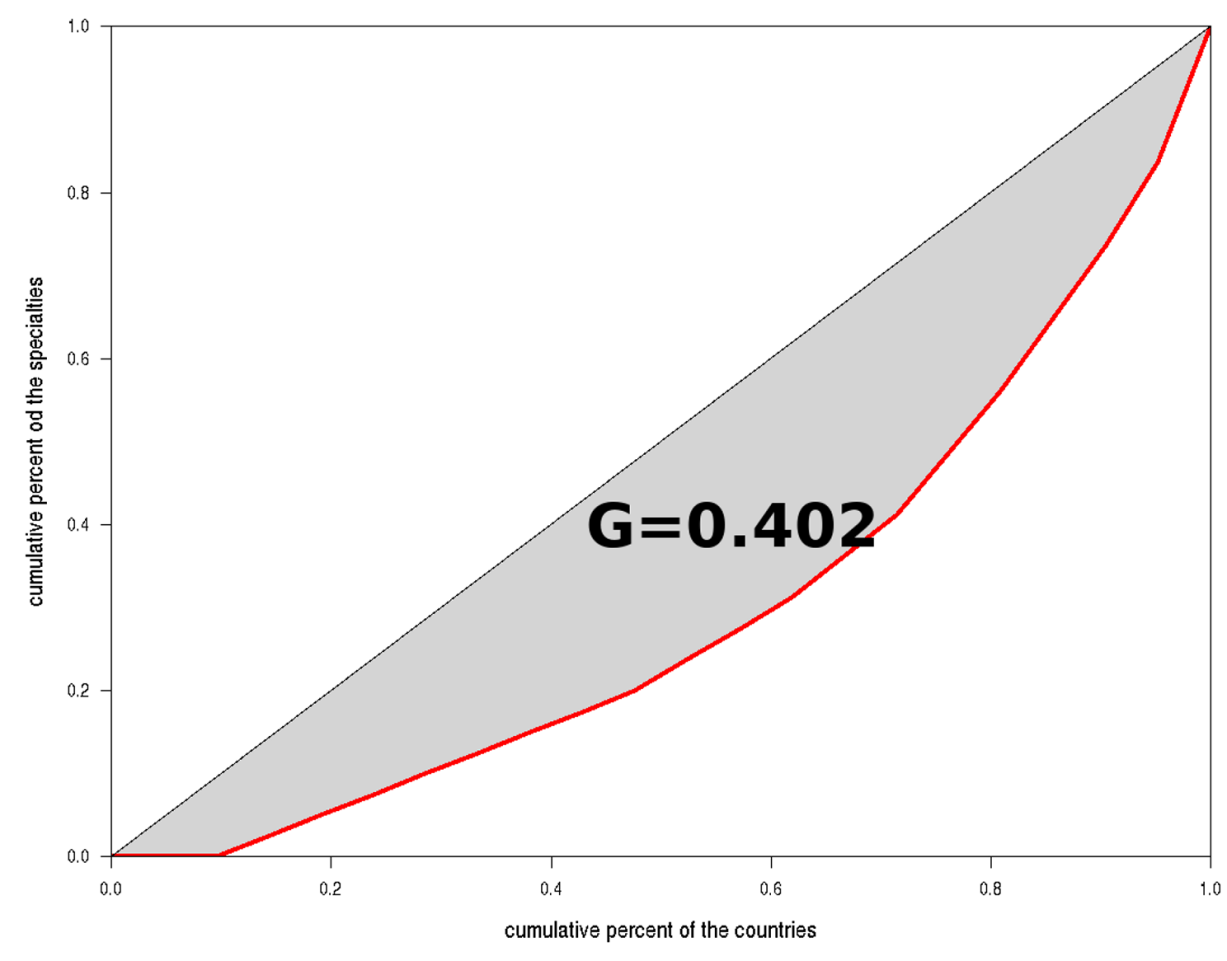
Lorentz curve representing the total global degree of inequality related to the number of dental specialties of the analysed countries

Figure 2 shows the Lorentz curve (in red) associated with the data of our study. The grey area represents dental inequality and its assessment using the Gini index was G = 0.402. So, the total global degree of inequality related to the number of dental specialties between the analysed countries, was 40.2%.

**Fig. 3:**
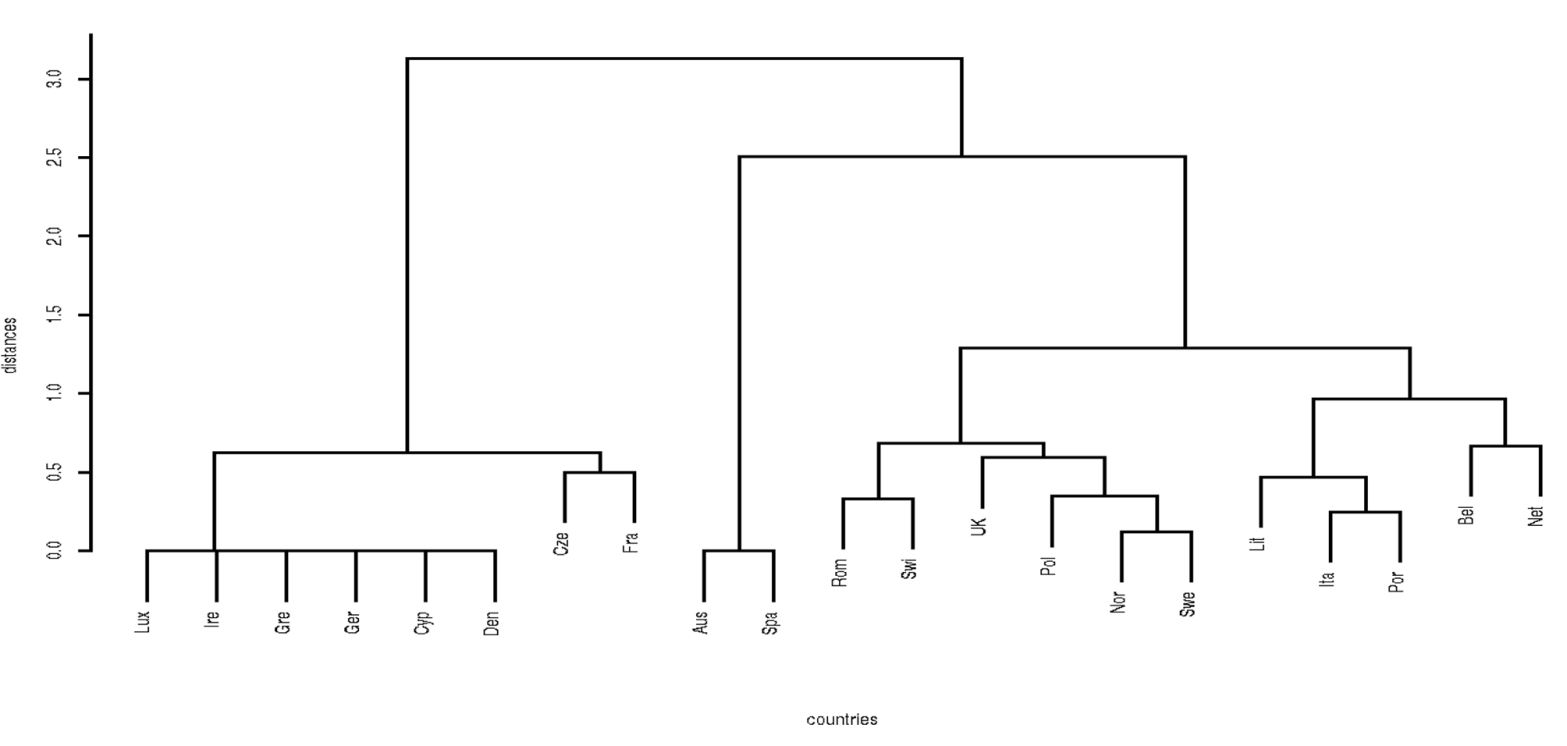
Cluster analysis of the number of dental specialties in the analysed countries

Cluster analysis differentiated three main groups of countries according to the number and type of dental specialties (Fig. 3). The associated dendrogram is displayed as a forked-line diagram where each fork, groups countries at its lower level. The height of each fork indicates the maximum distance between the countries that form it. In this way, interpreting our data, if we cut, for example, at distance 2 (the vertical axis) we would obtain three groups of homogeneous countries in terms of the number and type of specialties: Two large clusters of similar countries, the first one formed by Luxembourg, Ireland, Greece, Germany, Cyprus, Denmark, Czech Republic and France and the second one by Romania, Switzerland, the UK, Poland, Norway, Sweden, Lithuania, Italy, Portugal, Belgium and The Netherlands, and finally, a small group formed by only Austria and Spain. The lowest ranges, at zero distance, in the case of Luxembourg, Ireland, Greece, Germany, Cyprus and Denmark or Austria and Spain define those countries between which there are no differences at all.

## Discussion

The type of web-pages and documents that every state in the EES provides differ substantially depending on the way they organize their own health system, the type of annual reports that are written, the distribution of their documents or the visual aspect added to every public information provided. Nevertheless, whatever method is used, the information is accessible and clear inside the respective document or the Internet pages. A total of 21 countries having available and reliable data were included in our study [8–31].

Although the EES usually acts in a united and organized way, several main differences are found regarding the type and number of dental specialties all around this group of member states. It is important to highlight the high prevalence of two common-historical specialties, the first in being accepted and generally recognized, Orthodontics and Oral Surgery [1]. So, nowadays they appear in every country except in Spain [28] and Austria [8] (without any officially recognized dental specialty) and Belgium [9] (having recognized Orthodontics and Periodontology, but not Oral Surgery). The rest of the specialties are recognized in less than half of the countries studied in the EES.

A small group of official dental recognitions are less common, finding some of them like Oral Microbiology, Oral Pathology, Public Health or Special Care Dentistry that are only regulated as specialties in the United Kingdom [31], the country having the highest number. The rest of specialties such as Oral Medicine, Radiology and Restorative Dentistry are locally extended, existing exceptions like Stomathognathic Physiology, that is exclusively found in Sweden [29]; or Maxillofacial Surgery that is only officially recognized as a dental specialty in the Netherlands [22, 23] and in Lithuania [19, 20] (in this case, it accompanies Oral Surgery as two different dental specialties).

According to the number of officially recognized dental specialties quite a diverse variety was found. The number of dental specialties is really unbalanced, oscillating between 0 and 13 different specialties. Moreover, in some cases even the name and guidelines that every recognition serves is slightly different, which adds even more inequality and confusion between countries with the same type of officially recognized dental specialties [2, 3]. To quantify the degree of global inequality related to the number of dental specialties of the EES analysed countries plus the UK, the Lorentz curve and Gini index were applied.

Globally, in the countries of the EES there is an inequality with respect to the number of specialties of 40.2%. Luxembourg, Ireland, Greece, Germany, Cyprus, and Denmark have the same situation, having recognized only the two most frequent dental specialties. Austria and Spain are in a similar situation to each other, and they are an exception inside the EES with a lack of legal regulation that prevent them having officially recognized dental specialties. Spain, curiosly, has a lot of health specialties, but not in Dentistry (Medicine, Pharmacy, Nursing, Biology, Psychology or, surprisingly, Chemistry or Physics). Influencial factors could have had an impact in Spain, as frequent governmental changes (since 1978 there have been 15 different legislatures and 26 different Health ministers) that have been delaying the process; the more prevalent number of private dental practices (who do not require so much legal regulation, as is the case in other countries in the EES); a certain historical distance from dentists to dental specialties, or an unsuccessful professional management, could have tipped the scale against national recognition until now. Additionally, in both countries, dental specialties, particularly Oral Surgery, have been fought by physicians having the specialty of Maxillofacial Surgery.

The UK is the country having the highest number of dental specialties (13) and another 5 countries have more than 6 official specialties (Sweden, Lithuania, Norway, Poland and Romania), but the most of the EES countries count on just two specializations.

The recognition of dental specialties of the countries integrating the EES plus the UK has meant a benchmark in terms of increasing professional development. Apart from the problems associated with inequalities regarding the number or name of dental specialties, we must consider other quality defects caused by this scenario: Lack of access to officially recognized dental specialists, lack of specialized training, many restrictions to work abroad keeping the same job specialization and guarantees, or the difficulty to control the name of specialists countrywide. These are only some deficiencies that still need to be solved to recuperate the equalities and union that the EES should provide to every member state.

Our findings show the relevant dental specialties asymmetric situation existing in Europe. The results could be useful for both, dental professionals (i.e. in terms of conditions and freedom to move and work between countries across European countries, or protecting the public and the specialties themselves from unqualified clinicians) and the general population (i.e. regulating the quality in clinical care received by the population).

## Conclusions

The situation of dental specialties in the area of the EES plus the UK exhibits an unequal organization. Despite having some common directives that every country integrating the EES should follow, we can appreciate that actually it is not well implemented. Thus, the number and the name of officially recognized dental specialties across the member states is quite different and the total global degree of inequality related to the number of dental specialties of the analysed EES countries plus the UK is 40.2%, showing three different main clusters of countries with a similar pattern of dental specialties.

## Data Availability

All data generated or analysed during this study are included in this published article.
